# Breaking Free From MCHC Interferences? French‐Speaking Cellular Haematology Group (GFHC) Review of Causes, Rising Trends and Practical Solutions

**DOI:** 10.1111/ijlh.14536

**Published:** 2025-08-17

**Authors:** Sandrine Girard, Yaël Berda‐Haddad, Chantal Brouzes, Bouchra Badaoui, Agathe Boussaroque, Alexandre Janel, Bernard Chatelain, Véronique Baccini

**Affiliations:** ^1^ Haematology Biology Hospices Civils de Lyon Lyon France; ^2^ Haematology Biology University Hospital Timone, AP‐HM Marseille France; ^3^ Haematology Biology AP‐HP Hospital Necker France; ^4^ Haematology Biology AP‐HP Hospital Henri Mondor France; ^5^ Haematology Biology University Hospital Angers France; ^6^ Haematology Biology INOVIE GENBIO Clermont‐Ferrand France; ^7^ Haematology Biology CHU UCL Namur Yvoir Belgium; ^8^ Haematology Biology University Hospital Guadeloupe, Pointe‐à‐Pitre, Université Paris Cité France

**Keywords:** decision trees, GFHC, interferences, MCHC, recommendations

## Abstract

Mean corpuscular haemoglobin concentration (MCHC) is determined by the ratio of haemoglobin concentration to haematocrit. Managing increased MCHC presents significant challenges, mainly due to variations in analytical methods and pathophysiological conditions. Depending on the haematological analyser (HA), MCHC can be measured directly or calculated. It is important that all people involved in hematocytometry must identify and correct artefacts to ensure accurate erythrocyte parameters. In order to harmonise and standardise haematology practices in all laboratories, the French‐speaking Cellular Haematology Group (GFHC) has reviewed the interferences and pathophysiological situations that could increase MCHC, and the advice on how to manage cases of elevated MCHC. We will review current techniques, such as impedance and optical methods, for accurate determination of MCHC. We will also examine the interferences that can artificially increase MCHC; and the pathophysiological conditions responsible for such increases. Finally, we will present guidelines for the management of elevated MCHC, including strategies to bypass interferences and determine which erythrocyte parameters can be reliably reported, as well as the acceptable MCHC values for various pathophysiological variations.

## 1 Introduction

The management of red cell parameters in the laboratory is a sensitive topic [1, 2, 3]. In fact, erythrocytic parameters are interdependent, and they are subject to both analytical and pathological variations. Some parameters, such as the haemoglobin (HGB) concentration, are always measured, while others may either be measured or calculated [4]. In addition, the measured parameters can be affected by interferences, which must be identified.

Mean corpuscular haemoglobin concentration (MCHC) is commonly used as an analytical control because a high MCHC value indicates an analytical artefact [5]. However, certain pathological situations, such as red blood cell (RBC) disorders, result in elevated MCHC values due to changes in RBCs without them being inaccurate.

It is important to recognise the situations that can artificially increase the MCHC in order to implement solutions to correct the spurious parameters and/or ensure the accuracy of the RBC indices.

First, we will describe an overview of the techniques currently available. Then we will detail the interferences leading to these three situations: overestimation of HGB, underestimation of RBC count and underestimation of mean corpuscular volume (MCV). We will also develop special cases that represent the RBC diseases, neonates and hyperleukocytosis.

And finally, we will detail our recommendations for the management of various situations involving elevated MCHC and explain how the MCHC value should be checked, and the result reported. This document was produced by a working group on behalf of the French‐speaking Cellular Haematology Group (GFHC) consisting of eight experts in cellular haematology from university hospitals, general hospitals and private practices.

## 2 MCHC Evaluation

The reference method for assessing MCHC involves measuring HGB concentration via spectrophotometry and determining haematocrit (HCT) by centrifugation (spun HCT).

MCHC is calculated using the formula: MCHC = HGB/HCT [6].

While haematology analysers (HAs) are calibrated using this approach, it is important to recognise the limitations associated with these measurements. Spectrophotometric HGB can be affected by haemolytic, icteric or lipemic plasma leading to overestimations. Spun HCT is sensitive to factors like trapped plasma in RBCs, variations in plasma volume and in RBC characteristics (size, shape, deformability and fragility), resulting in imprecise operator‐dependent results.

HAs are calibrated with samples featuring normal HCT, so discrepancies are more pronounced when the RBC population is abnormal.

The higher the HCT is, the more trapped plasma there is, causing a greater difference between automated and spun HCT values, with an overestimation of HCT by the centrifugation method [7].

## 3 Different Technologies of HAs

### 3.1 Impedance Methods

#### 3.1.1 Sample Preparation

The sample preparation for RBC counting involves two key processes: sample acquisition and sample delivery. Both the patient sample and the reagents are delivered to the analysis chamber for preparation. While sample volumes and dilution methods may vary between suppliers, the final dilution is always quite high.

#### 3.1.2 Sample Measurement: Principle of Measuring Impedance Variation

Suspended cells in a conductive isotonic liquid are pulled through a calibrated orifice, concurrently with a direct electrical current, producing a change in impedance proportional to the volume of the cell traversing the orifice. For each cell lineage, the analyser sorts and classifies cells based on their volume [8, 9].

Normal RBC distribution follows a Gaussian curve with the most frequent volume (R‐MFV) defining the peak and aligning with MCV. R‐MFV is an additional RBC research parameter available in place of MCV and provides a better reflection of the true MCV in cases of interferences in RBC count by impedance method (RBC‐I).

To measure HGB concentration, the International Committee for Standardisation in Haematology (ICSH) recommends the DRABKIN method as the reference method [10], in which HGB is converted into cyanmethemoglobin (HGB cyanide). In HAs, a sample volume of whole blood is added to a hypotonic diluent to lyse RBCs; this step is usually accelerated by adding a non‐ionic detergent. Spectrophotometric absorbance of the HGB solution at a selected wavelength of light permits the HGB concentration determination. Due to the use of toxic cyanide in the DRABKIN method and its lack of automation, reagents for automated spectrophotometric methods have been developed for automated systems [11].

Therefore, RBC, HGB, MCV, HCT, mean corpuscular haemoglobin (MCH), MCHC and RBC distribution width (RDW) are either measured or calculated parameters, depending on HAs used [8, 9, 12] (Table 1).

**TABLE 1:** Methods for determining erythrocyte parameters based on impedance technology.

RBC	Always measured parameter	
HCT	Two different methods	
	Measured	Calculated
	Via the RBC pulse height detection method simultaneously with haemoglobin measurement	From RBC and MCV, using the following equation: HCT (L/L) = [RBC (T/L) × MCV (fL)]/1000
MCV	The amplitude of the pulse described paragraph 3.1.2 is proportional to the volume of the cell that produced it, thus determining the cellular volume *R‐MFV″ by the manufacturer Sysmex, represents the most frequent RBC volume and corresponds to the height of the distribution curve of impedance‐counted RBCs	If calculated, from the RBC and HCT, using the following equation: MCV (fL) = [HCT (L/L)/RBC (T/L)] × 1000
MCH	Always calculated from the RBC and HGB, using the following equation: MCH (pg) = [HGB (g/L)/RBC (T/L)]	
MCHC	Always calculated from the HCT and HGB, using the following equation: MCHC (g/L) = HGB (g/L)/HCT (L/L)	

Abbreviations: HCT, haematocrit; HGB, haemoglobin; MCH, mean corpuscular haemoglobin; MCHC, mean corpuscular haemoglobin concentration; MCV, mean corpuscular volume; RBC, red blood cell.

### 3.2 Optical Methods

RBCs and reticulocytes can be analysed using optical flow cytometry (RBC‐Op and RETIC, respectively) and/or light scattering technology. A test sample is aspirated, treated with a lysis and isotonic reagent to perforate leukocytes (WBC) membranes, and spheroidize RBCs and RETICs for optical analysis [13]. Hydrodynamic focusing ensures single cell passage. The biconcave shape of RBCs can deform, altering light scattering signals. Spherization creates homogeneous dielectric spheres that produce consistent signals according to Mie's theory [13] of light scattering.

HAs—Siemens Advia 2120i, Abbott CELL‐DYN Ruby, and Abbott Alinity h‐series—capture scattered light at multiple angles, improving discrimination between normal and pathological RBCs and permitting identification of microcytes, spherocytes, RBC fragments, giant platelets, and debris. RBC cytograms visualise RBC volume and HGB concentration (HGB‐Op), making it easier to assess cell size distribution and chromia variations. The Sysmex XN/XR Series and Mindray BC‐6800+ incorporate heated channels to reduce cold agglutinin interference [14].

RETIC properties are measured by scattering and lateral fluorescence after RNA staining with fluorochromes (e.g., oxazine 750, polymethine, asymmetric cyanine), differentiating RETIC maturity based on nucleic acid content.

High‐angle scattered light defines the optical HGB concentration, enabling simultaneous analysis of RBC volume and HGB. RBC HGB content—RBC‐He (Sysmex), CH RBC (Siemens Advia 2120i), and CHC (Abbott Alinity h‐series)—correlates with impedance MCH. HGB‐Op is derived from RBC HGB content and RBC‐Op, requiring recalculation of MCHC and MCH [5] or calculated from measured MCHC (Table 2). These optical parameters help to manage lipemic, icteric and haemolytic interferences that affect spectrophotometric HGB values and RBC issues like cold agglutinins (if heated channel used) and extreme microcytosis [15].

**TABLE 2:** Methods for determining erythrocyte parameters based on optical technology.

RBC‐Op	Detection and counting cells on the basis of light scattering.
MCV‐Op	Evaluation of RBC volume by light scattering.
HCT‐Op	Calculation based on MCV‐Op and RBC‐Op count using the following equation: HCT‐Op (L/L) = [RBC‐Op (T/L) × MCV‐Op (fL)]/1000
MCH‐Op	Based on *FSC* which correlates with both *cell size and HGB content in mature RBCs*
MCHC‐Op	Calculated from *MCH‐Op and MCV‐Op*, offering an indirect HGB concentration measure in using the following equation: MCHC (g/L) = [MCH‐Op (pg)/MCV‐Op (fL)] × 1000

Abbreviations: FSC, forward scatter signals; HCT‐Op, optical haematocrit; HGB, haemoglobin; MCHC‐Op, optical mean corpuscular haemoglobin concentration; MCH‐Op, optical mean corpuscular haemoglobin; MCV‐Op, optical mean corpuscular volume; RBC‐Op, optical red blood cells.

## 4 Situations Leading to Increased MCHC

First, we will describe the situations related to inappropriate pre‐analytical conditions or other biological situations leading to an overestimated HGB value.

We will then detail cases leading to a decrease in RBC count or MCV; finally, we will address specific situations where elevated MCHC is not necessarily due to interference, but rather due to underlying pathophysiological conditions (e.g., RBC diseases, neonates, hyperleukocytosis).

### 4.1 Pre‐Analytical Variables and HGB Overestimation

#### 4.1.1 Main Pre‐Analytical Variables

**FIGURE 1:**
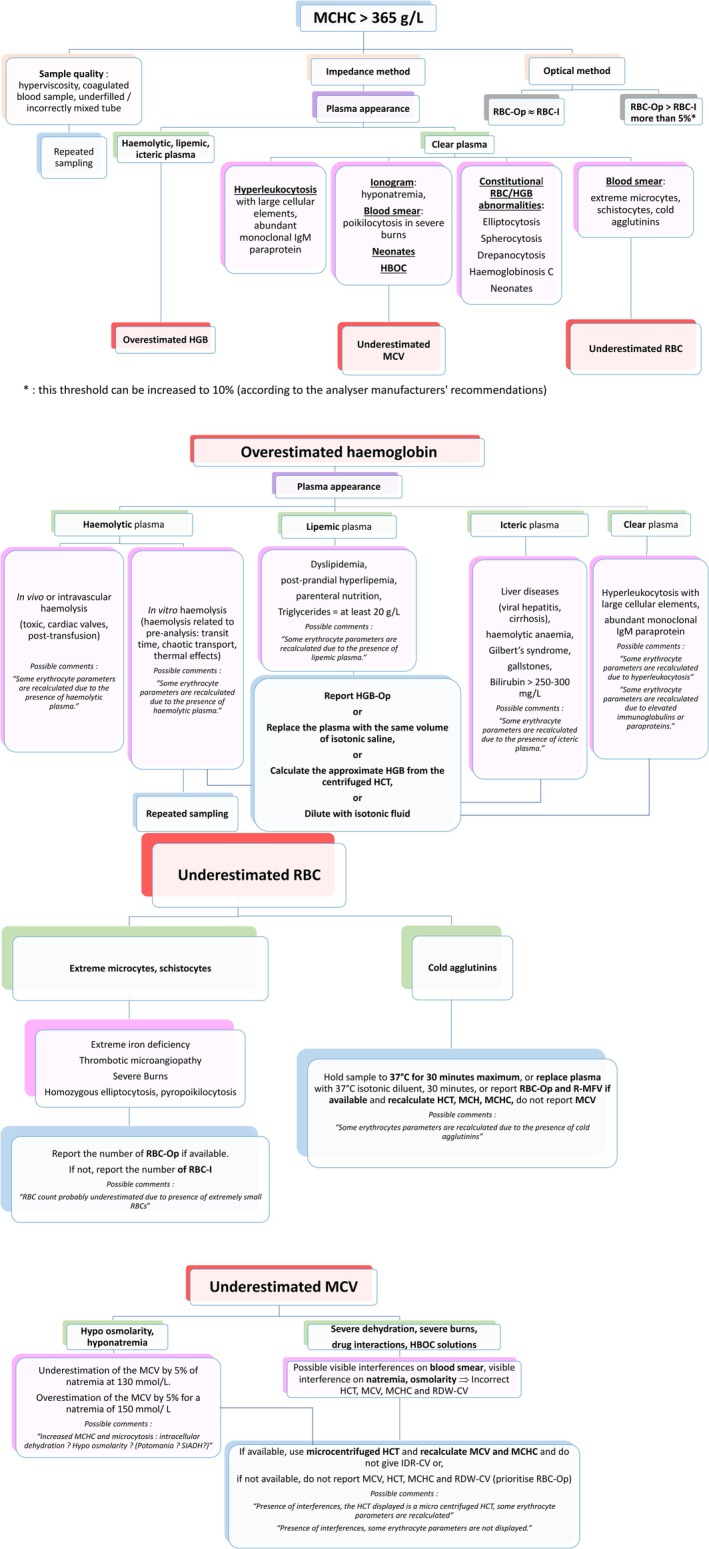
GFHC guidelines: Decision trees to manage MCHC increases. HBOC, haemoglobin‐based oxygen carrying; HCT, haematocrit; HGB, haemoglobin; HGB‐Op, optical haemoglobin; IgM, immunoglobulin M; MCH, mean corpuscular haemoglobin; MCHC, mean corpuscular haemoglobin concentration; MCV, mean corpuscular volume; RBC, red blood cell; RBC‐I, RBC with impedance count; RBC‐Op, RBC with optical count; RDW‐CV, red blood cell distribution width; R‐MFV, RBC most frequent volume; SIADH, Schwartz–Bartter syndrome/syndrome of inappropriate antidiuretic hormone.

– Capillary sampling can yield slightly different RBC indices, with MCHC often being lower under equivalent pre‐analytical conditions [8]. However, sequential pressure sampling can lead to oxidative cellular stress, reducing cell volume and increasing MCHC. In addition, excess EDTA (underfilled tubes) leads to RBC shrinkage, resulting in increased MCHC (Figure 1). This phenomenon occurs with both di‐ and tri‐potassium EDTA, though it is more pronounced with the latter [16].
– If a blood sample coagulates or is improperly mixed due to overfilling, the aliquot taken for the RBC count will be inadequate; the RBC count may be inaccurate (Figure 1).

#### 4.1.2 Main Interferences Causing Overestimation of HGB

HGB measurement can be interfered with by lipemic, icteric, or haemolysed plasma, leading to increased turbidity and spectral interference, which may result in overestimated HGB and falsely elevated MCHC.

This is particularly observed in cases of:

– Severe hypertriglyceridemia, either constitutional or acquired [10] and in cases of parenteral nutrition with lipid infusions [17]. Such interferences are typically seen at very high levels of hypertriglyceridemia (> 20 g/L). For lipemic plasma, especially following intravenous fat emulsions, a new sample should be taken and analysed remotely from the infusion.
– In patients with elevated bilirubin concentrations (> 250–300 mg/L), observed in several conditions like liver disease (e.g., viral hepatitis or cirrhosis), haemolytic anaemia, Gilbert's syndrome and gallstones [2, 17].
– In case of RBC destruction by excessive agitation (e.g., sudden transport or abrupt handling of the tube) or during difficult blood collection, syringe aspiration. This causes in vitro RBC destruction releasing HGB into the plasma, and resulting in a falsely elevated MCHC [1]. A new sample is then required; usually, free plasma HGB, measured at the same time as that contained in RBCs, is typically very low (< 0.2 g/L) and does not interfere. However, in cases of severe intravascular haemolysis, such as those caused by toxic agents, mechanical cardiac valves or post‐transfusion, free plasma HGB can become significant enough to disturb total HGB measurement [17], leading to erroneous results.

HAs typically dilute samples before HGB measurement, reducing but not always eliminating interferences. Methods are recommended for lipemic, icteric or in vivo haemolytic plasma. HGB‐Op can be reported instead of HGB from photometric measurement. If optical data are unavailable or insufficient, plasma can be replaced with an equal volume of isotonic saline, and the sample reanalysed. If available, microcentrifuged HCT can be used to estimate HGB. An alternative method to obtain reliable results is to dilute the blood specimen with an isotonic fluid (preferably the analyser diluent) and repeat the analysis (Figure 1). However, caution is advised to avoid errors in the dilution process or the correction of results based on the dilution performed.

– In cases of abundant monoclonal IgM paraprotein or cryoglobulins, some reagents can precipitate with IgM and interfere with HGB measurement despite a normal plasma appearance. This interference can mask true anaemia and lead to a falsely elevated MCHC. In this situation, HGB‐Op is used.

### 4.2 Spuriously Decreased RBC Count

Increased viscosity in blood samples can occur due to agglutinins, cryoproteins (e.g., cryoglobulins and cryofibrinogen), monoclonal immunoglobulins (IgM) or when RBCs are increased, such as in neonates or several disorders (such as polycythaemia, spherocytosis, hypochromic anaemia, sickle cell anaemia). In this case, partial gelation of the blood sample can mimic coagulated blood, causing irregular flow patterns in analysis chambers. This may lead to insufficient aspiration and underestimation of RBC count, resulting in a falsely elevated MCHC [6].

#### 4.2.1 Agglutinins

Agglutinins induce RBC clumping. When the operating temperature of the agglutinin is below 37°C (typically with a lower thermal optimum below 30°C, often around 4°C), it is termed a cold agglutinin; whereas, when the temperature exceeds 35°C (with a thermal optimum range of 35°C–40°C), it is termed a warm agglutinin. In both scenarios, upon reaching the thermal optimum, small aggregates of RBCs are counted as individual particles, leading to an underestimation of RBC count and falsely elevated MCHC. Conversely, larger RBC agglutinates are not considered in the RBC count, particularly when their size exceeds the maximum threshold for measuring RBCs (250–360 fL depending on the analyser).

Analysers operating near 37°C demonstrate reduced susceptibility to this interference. However, if the agglutinin titre is exceptionally high, even minor interference in RBC parameters may notably persist despite analytical conditions approximating 37°C. The subtle increase in MCV, at times within normal values, poses a challenge in detecting the presence of agglutinins, as MCHC remains at the upper limit of normal values.

In the optical method, when a heated channel is present, the RBC‐Op obtained can be reported in association with R‐MFV if impedance causes problems with RBC counting. HCT, MCH, MCHC, and reticulocyte count (Ret#) will be recalculated from these two new parameters [5] (Figure 1). If it is not sufficient, alternative methods have to be applied.

The observed anomalies vanish upon warming the sample (in an incubator or water bath at 37°C for 30 min) and then reanalysed. Alternatively, after centrifugation, replacing plasma with an equal volume of warm iso‐osmotic solution, a homogenisation and an incubation at 37°C for 30 min can be applied before complete blood count analysis. In rare cases where the agglutinin titre is exceptionally high and normalisation of the RBC count cannot be achieved even after warming the sample to 37°C, a new sample should be collected and kept at 37°C until analysis [18] (Figure 1).

The lack of correction after reheating to 37°C is the main difference between hot agglutinins, which are predominantly IgG and cause extravascular haemolysis, and cold agglutinins, which are predominantly IgM and cause intravascular haemolysis. To date, no standardized or reproducible guidelines exist for the detection and management of warm auto agglutinins in laboratories. Heat incubation is contraindicated, as elevated temperatures may enhance the thermal optimum of these autoantibodies, thereby increasing their activity. Confirmation of the warm nature of the agglutinin is typically achieved through elution testing performed during direct antiglobulin tests, as conducted by French Blood Transfusion Centres. Supporting arguments for the presence of a warm agglutinin include a non‐elevated MCHC despite classical disruption of RBC indices, as well as a complete lack of improvement in these parameters following incubation at 37°C (as also observed in some high‐titre cold agglutinin cases); and in some instances, worsening of these abnormalities, which further supports the diagnosis.

#### 4.2.2 Haemolysis

Significant in vivo haemolysis can persist in vitro (in the tube) and lead to a false decrease in RBC count and then a higher MCHC. This occurs in cases of post‐transfusion or toxic haemolysis [17]. The RBC count may be reported without any correction, but with a comment stating ‘haemolysed sample, probable underestimation of the RBC count’.

#### 4.2.3 Small RBCs

##### 4.2.3.1 Schistocytes/RBC Fragments Including Micro Spherocytes

The accuracy of RBC counts can be enhanced by certain HAs that use a specific reagent to isovolumetrically convert RBCs into spheres. Fragment size varies, often falling within the platelet counting range of many HAs. Counting platelets and RBCs in the same channel poses challenges in discriminating between small RBCs and large platelets in specific situations, particularly in cases of deep microcytosis with the presence of mechanically induced RBC fragments (schistocytes) or not (RBC fragments).

##### 4.2.3.2 Extremely Microcytic RBCs

Blood samples exhibiting significant microcytosis (particularly MCV < 50 fL) may contain RBCs that are too small (falling below the analyser's RBC count threshold) to be counted as RBCs. Their size matches the platelet count threshold, leading to their misclassification as platelets, particularly in the impedance method. Consequently, the RBC count is falsely low, but it often lacks clinical significance. Some analysers exclude particles with a volume below 36–40 fL (RBC fragments) or extremely microcytic RBCs (such as in severe burns), which fall below the discriminating threshold for distinguishing RBCs from platelets. This can underestimate the RBC count, but the impact is generally limited and typically does not exceed 5%–6%.

In both cases, RBC‐Op is likely to be more accurate and representative. If optical data are unavailable, RBC‐I can be reported with a comment (Figure 1).

### 4.3 Underestimation of MCV

Hyponatremia leads to intracellular dehydration, resulting in MCHC values exceeding 365 g/L, sometimes nearing 400 g/L. In these situations, an in vivo equilibrium occurs with RBCs in balance in a hypo‐osmotic environment. However, when analysed in the HA, the RBC encounters a medium with much higher osmolarity, leading to rapid cell dehydration and elevated MCHC that does not reflect the in vivo value. As an initial approach, measuring sodium levels allows assessing the patient's plasma osmotic status [19]. However, this sodium effect is not visible in vitro without the patient's in vivo osmotic equilibrium status [20]. This influence of sodium level is significant, as observations suggest that MCV may be underestimated by 5% for a sodium level of 130 mmol/L and overestimated by 5% for sodium levels at 150 mmol/L [19].

The influence of natremia on MCHC cannot be circumvented by current technologies. Some authors have proposed ‘normalising’ MCHC through correcting to an equivalent value for a ‘sodium 140 mmol/L’ [19]. However, without broader‐scale studies considering all solutes used by various providers, this approach seems impractical.

In cases of severe dehydration, MCV can be underestimated due to overall intracellular dehydration and increased intracellular HGB concentration in RBCs. However, due to often associated hypernatremia [21], the MCHC is usually only moderately increased; it is not associated with a prognostic impact.

Similarly, drug interactions or the use of haemoglobin‐based oxygen carrying (HBOC) solutions may lead to increased MCHC without clear reasons.

During these situations, HCT, MCV, MCHC and RDW become unreliable. RBC‐Op will be prioritised in association with spun HCT if available, authorising recalculation of MCV and MCHC. Otherwise, MCV, HCT and MCHC are cancelled (Figure 1).

### 4.4 RBC Diseases

In RBC diseases, MCHC is frequently increased, sometimes exceeding 380 g/L, primarily due to dehydration at the cellular level rather than pre‐analytical issues. This phenomenon is often seen in sickle cell disease, HGB SC disease and hereditary spherocytosis.

At steady state, RBC membrane permeability is minimal, and RBC volume is controlled by monovalent cation content.

Key mechanisms for RBC cation leakage include:

– Na^+^ K^+^ ATPase pump: This pump maintains in RBCs high intracellular potassium (K^+^) levels (around 140 mEq/dL) and low intracellular sodium (Na^+^) levels (around 10 mEq/dL) [22]. An influx of Na^+^ over K^+^ leads to overhydration and decreased MCHC; while the opposite causes dehydration and increased MCHC.
– PIEZO channel: PIEZO1 is a mechanosensitive ion channel in the RBC membrane [23]. When activated, it allows calcium influx, which triggers K^+^ loss via the Gardos channel, leads to RBC dehydration and increased MCHC [24].
– KCC (potassium chloride cotransport): This volume‐sensitive pathway facilitates K^+^, chloride, and water loss, reducing RBC volume and increasing MCHC.
– P^sickle^ a deoxy‐dependent non‐selective ion and small solute permeability pathway that mediates reversible increases in permeability in sickle cells; P^sickle^ allows calcium and other cations entry in RBCs during hypoxia, triggering the Gardos channel [25] and KCC [26].

In sickle cell disease, dense sickle cells can reach MCHC levels of 500 g/L, with 40% of sickle RBCs exhibiting concentrations ≥ 380 g/L.

Concerning the guidelines for handling MCHC elevation in the case of RBC diseases:

– Known RBC disease: In these patients, the elevated MCHC should be evaluated in conjunction with a blood smear and consideration of other potential MCHC interferences (e.g., plasma appearance). If no additional interferences are detected, the MCHC value can be reported directly. Nevertheless, the consensus is that the maximum tolerated MCHC value should be 390 g/L.
– Unknown RBC disease: When a disease is suspected but not confirmed, the approach differs slightly. First, eliminate common MCHC interferences; then examine the blood smear and other relevant biological parameters such as Ret# and an ionogram. If necessary, collaborate with the clinician to establish a diagnostic protocol which may include advanced tests such as HGB electrophoresis, EMA test, ektacytometry or molecular biology assessments. In both cases, reviewing RBC morphology and clinical context is essential for accurate diagnosis of MCHC changes.

### 4.5 Neonates

Neonatal RBCs have distinct deformation characteristics and internal viscosity that are related to their MCHC. Unlike adult RBCs, foetal and neonatal RBCs are larger, have a shorter lifespan, possess altered shapes and deformability and contain more foetal HGB (HbF) [27].

Neonatal RBCs have a 21% increase in volume, 13% more surface area, and an 11% larger diameter compared to adult RBCs [28]. Newborns often show mild anisopoikilocytosis. Their RBCs are also less deformable and more fragile. Commonly observed abnormalities in newborns, especially in premature infants, include macrocytes, pitted cells, echinocytes, spherocytes and stomatocytes. Some researchers propose that neonatal RBCs may be bowl‐shaped or spherical rather than the typical biconcave disc shape.

The RBC count is lower in premature neonates, with a lifespan of 35–50 days, compared to 0–90 days for full‐term neonates. HGB and HCT levels are also lower, while MCV is higher in preterm infants [29]. In the initial hours after birth, plasma movement increases RBC count and HCT, influencing blood viscosity. Hyperviscosity occurs in 5% of infants and 18% of those small for gestational age [30]. Abnormal HCT or HGB levels vary with gestational age; those under 28 weeks have about 10 points lower HCT and a mean HGB 33 g/L lower than preterm and term neonates [31]. MCHC is lower in neonates than in adults and is not significantly affected by gestational age, showing a substantial increase in the first 5–6 weeks [28]. The average MCHC is similar across full‐term and premature infants and adults, around 340 ± 10 g/L. High MCHC can be a normal variant in the first week and relates to RBC size and HCT value [32, 33].

So, we suggest accepting MCHC results above 365 g/L during the neonatal period (birth to 30 days); however, after 30 days, such increases are concerning and require investigation. Based on recent clinical reference values, we recommend a threshold of 390 g/L, as the maximum MCHC for newborns from birth to 1 month is 389 g/L [34].

In neonates and young children, blood can be collected from various sites, including arterial puncture, catheterization of umbilical vessels or peripheral veins and finger stick punctures. HGB, HCT and RBC values are usually higher in capillary blood compared to venous or arterial samples, with significant differences noted in the neonatal period [35]. In addition, the MCV is lower in capillary blood, likely due to haemoconcentration. These factors may lead to an overestimation of MCHC in capillary sampling.

Neonates may show lower HCT levels with HA, potentially causing overestimation of MCHC [7]. Measured HCT can differ from spun HCT by 1%–3% to up to 6% in specific disorders [36]. Experiments with radio‐iodinated serum albumin have revealed that discrepancies in HCT measurements are mainly due to plasma entrapment during centrifugation [37, 38]. In cases of elevated MCHC, some labs use a two‐step method: measuring HCT via centrifugation and then recalculating the MCHC. This often results in higher HCT values than automated methods, aiding MCHC normalisation. However, spun HCT has low accuracy and can yield higher values if centrifugation is insufficient; whereas automated methods offer more accurate and stable results.

When various techniques, such as alternative centrifugation and impedance/optic methods, are applied to the same patient, consistent discrepancies arise. To prevent confusion, it is best to use either centrifugation or automated methods consistently for all samples from a patient during hospitalisation and follow‐up.

### 4.6 Hyperleukocytosis

Major hyperleukocytosis can interfere with HGB measurement and RBC count; thus, affecting RBC indices.

In cases of hyperleukocytosis caused by an increase in small cellular elements such as in chronic lymphocytic leukaemia (CLL) and acute lymphoblastic leukaemia (ALL), interference primarily affects RBC counting (RBC‐I) and the MCV measurement by the impedance method. In such situations, WBCs are falsely counted as RBCs, inducing overestimation of RBC‐I and an artificial increase in MCV and HCT [39]. On the RBC impedance curve, a double population can sometimes be observed, triggering alarms. As a result, MCH and MCHC may appear to be lowered due to the artefactual overestimation of RBC‐I. In this case, optical parameters or a dilution to reduce the interference are used to correct the false MCHC, as described in agglutinin interference (Figure 1).

When hyperleukocytosis is related to an increase in large cellular elements, such as in chronic myeloid leukaemia (CML) with myelemia, or acute myeloid leukaemia (AML) with large blasts and/or granular cytoplasm, the WBC count could interfere with HGB measurement by spectrophotometry but does not affect RBC‐I or MCV [39]. These large cells are automatically excluded from the RBC counts, as they exceed the upper limit of the RBC impedance curve.

Lysed WBCs release cytoplasmic content, especially in cells with granular content, leading to overestimated HGB, which correlates with the degree of hyperleukocytosis.

Therefore, while RBC‐I and MCV remain accurate, the HGB value is artificially high, causing an increased MCH and MCHC. In this case, HGB‐Op or dilution can be used to correct this false MCHC, as described in plasmatic interference (Figure 1).

## 5 Technical and Biological Validations of MCHC: GFHC Guidelines (Decision Trees)

Figure 1 summarises the main situations leading to an increased MCHC and proposes decision trees for each situation.

## Author Contributions

All authors contributed to the preparation, reviewing and revision of the manuscript. All authors prepared the article in their personal capacity and not as an official representative or otherwise on behalf of a sanctioned government.

## Ethics Statement

The authors have nothing to report.

## Consent

The authors have nothing to report.

## Conflicts of Interest

The authors declare no conflicts of interest.

## Data Availability

Data sharing is not applicable to this article as no new data were created or analysed in this study.
